# Stage-sensitive potential of isolated rabbit ICM to differentiate into extraembryonic lineages^†^

**DOI:** 10.1093/biolre/ioaf157

**Published:** 2025-07-22

**Authors:** Katarzyna Filimonow, Anna Chołoniewska, Jan Chołoniewski, Zofia E Madeja, Katarzyna Barłowska, Joanna Grabarek, Elżbieta Wenta-Muchalska, Berenika Plusa, Anna Piliszek

**Affiliations:** Department of Experimental Embryology, Institute of Genetics and Animal Biotechnology, Polish Academy of Sciences, Jastrzębiec n/Warsaw, Poland; Department of Experimental Embryology, Institute of Genetics and Animal Biotechnology, Polish Academy of Sciences, Jastrzębiec n/Warsaw, Poland; Department of Neurogenetics and Functional Genomics, Mossakowski Medical Research Institute, Polish Academy of Sciences, Warsaw, Poland; Faculty of Physics, Center of Excellence for Complex Systems Research, Warsaw University of Technology, Warsaw, Poland; Faculty of Veterinary Medicine and Animal Science, Department of Genetics and Animal Breeding, Poznan University of Life Sciences, Poznan, Poland; Department of Experimental Embryology, Institute of Genetics and Animal Biotechnology, Polish Academy of Sciences, Jastrzębiec n/Warsaw, Poland; Division of Developmental Biology & Medicine, University of Manchester, Manchester, United Kingdom; Department of Experimental Embryology, Institute of Genetics and Animal Biotechnology, Polish Academy of Sciences, Jastrzębiec n/Warsaw, Poland; Division of Developmental Biology & Medicine, University of Manchester, Manchester, United Kingdom; Department of Experimental Embryology, Institute of Genetics and Animal Biotechnology, Polish Academy of Sciences, Jastrzębiec n/Warsaw, Poland

**Keywords:** trophectoderm, preimplantation embryo, inner cell mass, blastocyst, differentiation, rabbit

## Abstract

In the course of mammalian development, the initial state of totipotency must be lost to allow the acquisition of specific cell fates. The first differentiation event results in the formation of trophectoderm (TE) and the inner cell mass (ICM). In the mouse embryo, the cell fate of these two compartments is set quickly after blastocyst formation. However, recent reports suggest that the plasticity of these two lineages might be extended in species other than the mouse. Here, we investigated how the cellular plasticity of early mammalian embryos relates to developmental time scale and changes in gene expression using rabbit isolated ICMs. We studied the dynamics of rabbit blastocyst formation using time-lapse imaging and identified GATA3 as an early marker of rabbit TE and CDX2 as a marker of fully formed TE. We then analysed the developmental potential of rabbit ICMs isolated by immunosurgery and subsequently cultured in vitro. ICMs originating from early- to mid-blastocyst stage embryos are able to re-form a blastocyst-like structure, with a functional TE, and an ICM containing both SOX2-positive epiblast cells and SOX17-positive primitive endoderm cells. We further observed that rabbit ICMs isolated from later blastocyst stages lose the ability for TE specification, instead forming a halo-like cavity with an outer layer of SOX17-positive cells. Our data indicate that in mammalian embryos, the potential for TE differentiation gives way to the formation of a different type of extraembryonic epithelial layer, suggesting a potential common mechanism of pluripotency restriction between eutherian mammals.

## Teaser

Rabbit ICM retains potential to differentiate into extraembryonic TE lineage past mid-blastocyst stage.

## Graphical Abstract

**Graphical Abstract:**
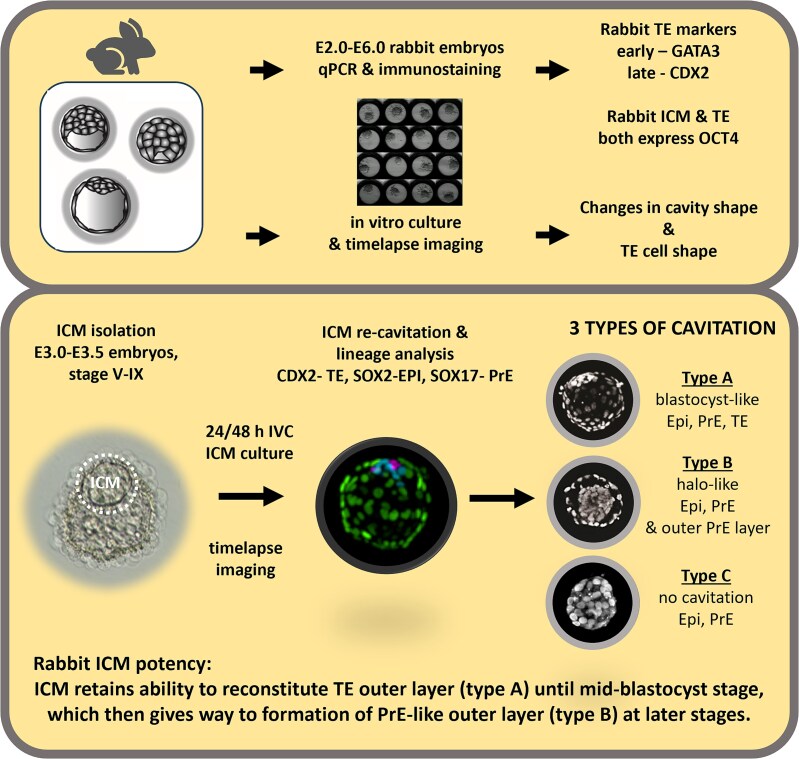

## Introduction

The early stages of embryonic development of eutherian mammals are devoted to differentiation of the first cell lineages—pluripotent epiblast (Epi), and extraembryonic primitive endoderm (PrE) and trophectoderm (TE). TE is the first cell lineage specified during mammalian ontogenesis. Proper differentiation of TE is a prerequisite for supporting the pregnancy, as TE is responsible for embryo implantation in the uterus and forms the embryonic part of the placenta (reviewed in [1]). Placental-related disorders affect around one-third of human pregnancies [2], and TE quality and cell number can be used as effective predictors of successful pregnancy in human assisted reproductive technologies [3, 4]. Despite significant knowledge about TE lineage specification in the mouse, data concerning this first differentiation event in other mammalian species, including human and domestic animals, are still limited. Importantly, a growing body of evidence indicates that notable differences exist in early lineage specification between mouse and other eutherian mammals [5–7] (reviewed in [8]). Embryos of human or primate origin are not easily available for research due to ethical concerns and the lack of easily accessible material. In addition, many experimental approaches are not applicable when using non-rodent systems and embryological studies in both primates and large domestic animals mainly rely on in vitro embryo production (IVP). Even the best IVP conditions are suboptimal when compared to the in vivo environment, and several reports show a significant influence of in vitro culture conditions on lineage allocation and lineage-specific gene expression in mammalian preimplantation embryos [9–11]. Therefore, it is critical that important developmental processes, such as TE specification, are studied in both in vitro and in vivo–derived embryos.

In mouse embryos, specification of the TE lineage from the outside cells of the morula is regulated by the expression of several transcription factors, including Caudal-related homeodomain transcription factor (CDX2) [12] and GATA-binding protein 3 (GATA3) [13, 14]. *Cdx2* gene expression in the mouse is already detected prior to cavitation, and at the blastocyst stage, it becomes restricted to TE cells [12]. CDX2 is also a specific TE marker in non-rodent mammals, including human, pig, cattle and rabbit [15–21]. However, in contrast to the mouse, in most of these species, CDX2 expression has not been confirmed prior to cavitation. GATA3 is also associated with TE fate specification in the mouse [13, 14]. GATA3 is co-expressed with CDX2 in mouse TE, and it is capable of inducing TE specification in mouse embryonic stem cells [14]. GATA3 has also been shown to be a specific TE marker in horse [22], cattle [23–25], and human embryos [23, 26].

At the initiation of embryonic development, individual cells (blastomeres) are totipotent and equal in their developmental potential [27–30]. This early totipotency has to gradually give way to the differentiation of the specific cell lineages and tissues, in order for the embryo to develop correctly. In the mouse, TE versus inner cell mass (ICM) cell fate becomes mostly restricted shortly after cavitation [31–35]. In contrast, human and bovine embryos retain high plasticity up to Day 6 of development [26, 36]. In cattle, ICMs isolated from Day 6 blastocysts are able to regenerate the TE layer, forming blastocyst-like structures that support full-term development [36], whereas human naïve epiblast can regenerate TE cells [26]. Currently, it still remains to be resolved whether a shorter period of ICM plasticity in the mouse represents a significant deviation from other mammals [26, 31–36].

To further understand these interspecific differences in TE versus ICM lineage specification during mammalian development, we looked for a model organism that allows for the analysis of in vivo fertilized embryos as well as experimental manipulation. Lagomorphs (including rabbits) have been reported to be genetically closer to Primates than Rodents are [37, 38], and the rabbit postimplantation embryo develops as a flat, bilaminar disc, similarly to many mammals, including humans [39]. Rabbit in vivo fertilized embryos are easily available for study, which allows to avoid any potential anomalies in development induced by the in vitro fertilization procedure. Here, we present analysis of TE-associated transcription factors expression and their spatio-temporal localization at the consecutive stages of rabbit blastocyst development in vivo, as well as time-lapse imaging-based examination of rabbit TE morphogenesis. We further investigate rabbit ICM commitment and potency by analyzing the regenerative potential of isolated rabbit ICMs. Our study shows that although TE-associated transcription factors are conserved among mammals, their regulation might significantly differ, especially between rodents and non-rodent species. We also reveal notable differences in the early lineage differentiation potential between species, evidenced by the delayed differentiation of rabbit ICM compared to the mouse.

## Materials and methods

### Animals

Rabbits (*Oryctolagus cuniculus*, Popielno breed) were maintained under a 14-h light/10-h dark cycle in the animal facilities of the Institute of Genetics and Animal Biotechnology of the Polish Academy of Sciences (IGAB PAS) according to the national and institutional guidelines. Experimental procedures were approved by the Second Local Ethics Committee, Warsaw, Poland (permission numbers: WAW2/183/2018 and 31/2012).

### Embryo collection and culture

Embryos were derived from females under general anesthesia or from the dissected reproductive tract of sacrificed animals following timed natural matings. Embryos were collected by flushing the oviduct (1–3 days post coitum, dpc) or uterus (4–6 dpc) of donor females with pre-warmed medium (TCM-199 + 10% fetal bovine serum (FBS), Sigma). Where indicated, embryos were cultured in vitro in drops of RDH medium (RPMI:DMEM:Ham’s F10, Life Technologies, at 1:1:1) supplemented with 0.3% bovine serum albumin [40], under mineral oil, in a humidified incubator, at 38.5°C, 5% CO_2_ in air. Before in vitro culture, embryo coats were pre-digested by 45 s incubation in 0.5% pronase, followed by 30 min incubation in TCM-199 + 10% FBS medium.

### Immunosurgery

Immunosurgery was performed to isolate the ICMs from the rabbit blastocyst by removing the TE layer [41]. Prior to immunosurgery, embryo coats were pre-digested as indicated earlier and then removed mechanically. Immunosurgery was performed by 30 min incubation in anti-rabbit serum (20% in TCM-199 + 10% FBS medium; 091 M4751, Sigma), followed by 15–20 min incubation in complement solution (20% in TCM-199 + 10% FBS medium; 234 395, Millipore). Lysed cells were removed mechanically by pipetting.

### Multifluorescent microspheres labelling

To evaluate the efficiency of immunosurgery we labelled intact blastocysts with fluorescent microspheres [42]. To this end, blastocysts were washed in TCM-199 medium (without serum or BSA) and incubated for 1 min with Flouresbrite Multifluorescent microspheres (0.2 μm; 1:100; Polysciences USA). After rinsing in TCM-199 medium, blastocysts with fluorescently labelled TE were subject to immunosurgery [43], and subsequently imaged to confirm the successful removal of TE (Figure S1).

### ICM culture

The isolated ICMs were cultured in the RDH medium supplemented with 0.3% bovine serum albumin, under mineral oil, in a humidified incubator, at 38.5°C, 5% CO_2_ in air (as indicated for whole embryos). ICMs were cultured under the PrimoVision time-lapse system (Vitrolife) for brightfield time-lapse imaging for 24 or 48 hours.

### Immunostaining and labelling

Embryos and ICMs were fixed in 4% paraformaldehyde in PBS with 0.1% Tween-20 (Sigma) and 0.01% Triton X-100 (Sigma) for 20 min at room temperature. Embryonic coats were removed mechanically after fixation [44] unless otherwise indicated. Fixed embryos and ICMs were immunostained as previously described [45]. Briefly, embryos were permeabilized in 0.55% Triton X-100 (Sigma) solution in PBS for 20 minutes, subjected to quenching unreacted aldehydes in NH_4_Cl for 10 minutes, and blocked in 10% donkey serum (Sigma) for 40 minutes. Embryos were incubated with primary antibodies overnight at 4°C. Primary antibodies were used at a 1:100, except anti-SOX2 antibody (abcam, 1:200). Alexa Fluor-conjugated secondary antibodies (Invitrogen: donkey anti-mouse Alexa 488, donkey anti-rabbit Alexa 647, donkey anti-goat Alexa 568) were used at 1:500. Primary antibodies are listed in Table 1. DNA was visualized using Hoechst 33342 (10 μM, Life Technologies, H3570).

**Table 1:** List of primary antibodies used.

Target	Host	Company	Cat. Number	RRID
CDX2	Mouse	BioGenex	MU392A-UC	AB_2923402
GATA3	Mouse	BioLegend	653801	AB_2561776
	Mouse	BioLegend	653809	AB_2563217
	Goat	R&D systems	AF2605	AB_2108571
OCT4	Goat	R&D systems	AF1759	AB_354975
SOX2	Rabbit	Abcam	ab97959	AB_2341193
	Goat	R&D systems	AF2018	AB_355110
SOX17	Goat	R&D systems	NL1924R	AB_2195645
	Goat	R&D systems	AF1924	AB_355060
GATA6	Goat	R&D systems	AF1700	AB_2108901
	Rabbit	Cell Signaling technology	5851	AB_10705521

For membrane labelling, in vivo-derived live embryos were labelled with vital dye FM4–64 (Invitrogen) at a dilution of 1/10 in TCM-199 medium for 30 minutes at 38.5°C, and immediately imaged under confocal microscope.

### Imaging and image analysis

Embryos were placed on a glass-bottom dish (Thermo Fisher) and three-dimensional (3D) imaging was performed using an A1R Nikon inverted confocal microscope. Embryos were imaged in their entirety with a 20x objective using Z-stacks of 2 μm thickness.

Analysis of images was performed using IMARIS software (Bitplane AG). For cell number count, nuclei were identified using the “spot” option with an estimated diameter of 7–10 μm. The total cell number was based on Hoechst chromatin staining, and the number of cells positive for each transcription factor was based on appropriate immunofluorescent staining. The number of nuclei identified by IMARIS was confirmed manually. 3D confocal images were created by maximum intensity projection using the IMARIS “volume” option.

Analysis of fluorescence intensity levels was also based on the spot detector function. To account for signal attenuation (i.e., the gradual reduction in fluorescent signal along the Z-plane of the embryo), OCT4 and GATA3 intensity values were normalized by dividing them by the nuclear signal intensity (Hoechst staining) [46]. For each embryo, the arithmetic mean of the normalized OCT4 and GATA3 signal was calculated separately for the TE and ICM. Next, the ICM-to-TE ratio was computed for each embryo, and the mean of this ratio across all embryos was determined for each developmental stage.

### Embryo collection for gene expression analysis

In-vivo-obtained rabbit embryos were collected at successive developmental stages at 2.0 (morula, stage IV), 3.0 (late morula, non-cavitated stage V-VI), 3.25 (early blastocyst, cavitated stage VI-VIII), 4.0, 5.0, and 6.0 dpc (blastocyst). For each developmental stage, we collected six independent samples, each sample containing four embryos. The selected material was placed in a minimal volume of PBS in 1.5 ml tubes (low binding, Eppendorf), snap-frozen in liquid nitrogen and stored at −80°C.

### RNA extraction and cDNA synthesis

Total RNA was extracted with the High Pure miRNA Isolation Kit (Roche Diagnostics) following the manufacturer’s protocol, as previously described [20, 45]. RNA quality and concentration were measured using NanoDrop c2000 (Thermo Scientific). For each sample, the reverse transcription reaction was performed from 100 ng of total RNA. cDNA synthesis was performed with the Transcriptor High Fidelity cDNA Synthesis Kit (Roche Diagnostics) following the manufacturer’s protocol. The samples were stored at −20°C.

### Quantitative real-time PCR reaction

Quantitative PCR (qPCR) was performed on a Roche Light Cycler 96 instrument. Calculations of gene expression level were based on the standard curve method (with series of 10 fold dilutions of known concentrations) with three reference genes: *H2AFZ*, *HPRT1* and *YWHAZ* [47]. The relative mRNA content was calculated to the normalized mean transcript level of the reference genes. Each sample was analyzed in triplicate, with all of the primer sets chosen for the experiment. The primer pairs were designed to span the introns. The reactions were carried out as previously described [20, 45]. Product specificity was confirmed by melting-point analysis and agarose gel electrophoresis.

### Statistical analysis

Data processing and visualizations were done in a Jupyter notebook using the pandas 1.4.4, Matplotlib 3.7.2 and seaborn 0.12.2 Python libraries. The expression levels of *CDX2*, *GATA3*, and *OCT4* in rabbit embryos between consecutive stages of development were compared using the Kruskal-Wallis test (SciPy 1.10.1). Subsequent pairwise comparisons were conducted using the Conover test with a two-stage FDR correction (scikit-posthocs 0.7.0). The statistical analysis of the ICM-to-TE fluorescence intensity ratio of OCT4 and GATA3 across developmental stages was performed using the Kruskal-Wallis test. Subsequent pairwise comparisons were performed using the Dunn test with Holm correction to uncover specific statistically significant distinctions.

## Results

### Dynamic changes of embryo morphology during preimplantation rabbit development

**Figure 1:**
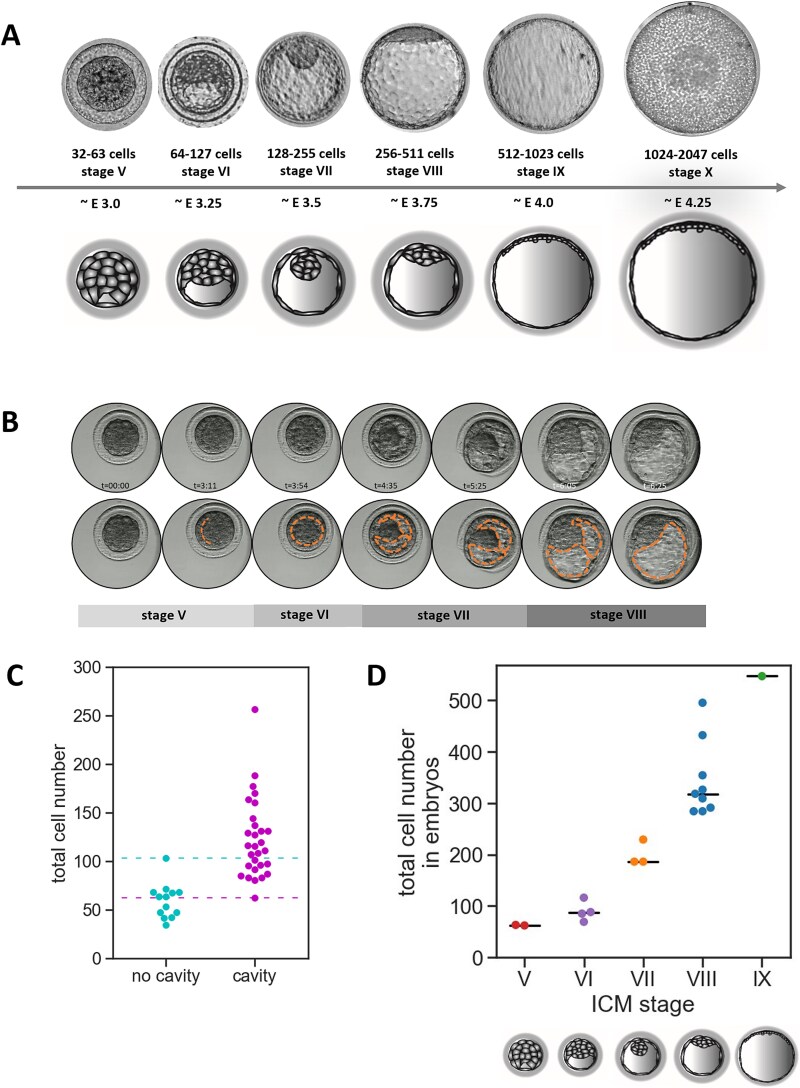
Dynamic changes of embryo morphology during rabbit blastocyst development. (A) Morphological landmarks are correlated with the cell number in E3.0–E4.25 rabbit embryos in vivo (embryos not to scale). Staging system based on total cell number. Presented timing (E3.0-E4.25) is approximate. (B) In vitro development of rabbit blastocyst. Selected sections from the Primo Vision time-lapse system. Orange dotted line denotes the boundaries of the blastocyst cavity. (C) Cell number in rabbit embryos at the time of cavitation. Dashed lines denote minimum and maximum cell number values for E2.75–E3.25 non-cavitated and cavitated embryos, respectively. (D) Rabbit embryo staging system based on cell number correlated with staging based on morphology. Calculation of cell number in embryos in vivo categorized to each stage based on the morphology.

**Figure 2:**
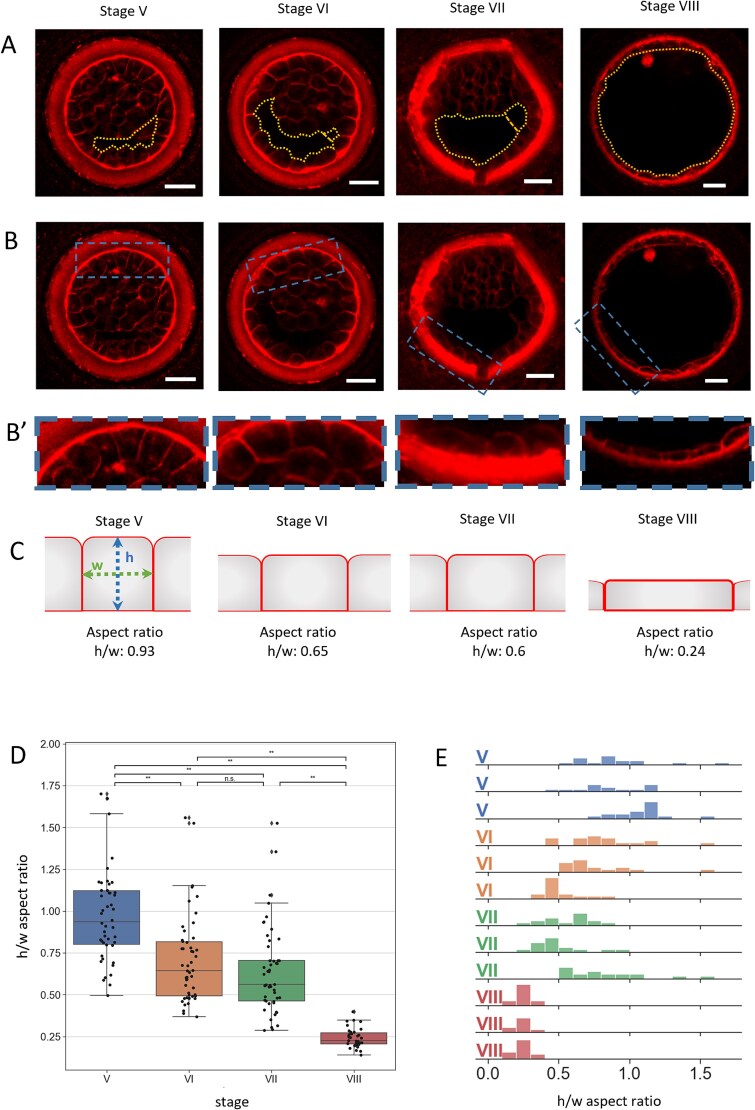
Changes of TE cell aspect ratio during rabbit blastocyst development. (A, B, B′) Live rabbit embryos at consecutive stages of blastocyst development, labelled with fluorescent lipophilic membrane dye, FM4–64; blastocoel cavity outlined with yellow dotted line. Scale bar- 50 μm. (B′) Magnification of TE section. (C) Corresponding schematic of TE cell aspect ratio measurements. (D) Box plots representing height to width aspect ratio of TE cells at consecutive stages of blastocyst development; Kruskal–Wallis statistical test; ^**^ = p < 0.001. (E) TE cells height to width aspect ratio in individual embryos—each row represents one embryo. Each bar represents the incidence of a particular aspect ratio in the embryo.

We have previously established that a specific pattern of transcription factor expression in rabbit preimplantation embryos is not correlated with the time post coitum per se, but rather with the total cell number of the embryo [45]. Our previous research also suggested that different blastocyst geometries are acquired during the consecutive stages of blastocyst maturation. To confirm that rabbit blastocyst indeed progresses through the specific geometries, and to gain more insight into the morphogenic processes driving this progression, in the current study, we analyzed the changes in the morphology of rabbit embryos during blastocyst cavity formation and expansion (Figure 1A, 1B, Movie S1). To this end, in vivo fertilized embryos were collected at consecutive stages of blastocyst development (E3.0 to E4.25; Figure 1A, C, D), or collected at the late morula stage on Day 3 of development, and time-lapse imaged during the 24-hour in vitro culture (n = 16) (Figure 1B). We paid specific attention to the changes in shape and size of the ICM, TE, and the cavity, in order to confirm the timely progression of the morphological landmarks (Figure 1A, B, Figure 2, Movie S1). Our observations can be summarized as follows: initiation of cavity formation becomes first apparent as a U-shaped slit between the outer and the inner cells. The slit then expands into an ellipsoid cavity, with ICM located at one of the poles (stage VI). Next, in stage VII blastocysts, the cavity becomes more crescent-shaped, while multiple cytoplasmic bridges can be detected between TE and spherical ICM (visualized in detail in Figure S2 and movie S1). At stage VIII, the cavity becomes nearly spherical, and ICM becomes more flattened against one pole of the cavity, although still clearly distinguished as a thickened area. At stage IX, the blastocyst cavity is spherical and ICM becomes flattened against polar TE. At later stages of rabbit preimplantation development embryos retains the same shape, and differ only in size, as blastocyst diameter further increases. Embryos obtained in vivo at embryonic day (E) 2.75–3.25 (n = 42), represented late morula and early blastocyst stage. Initial stages of the cavitation were observed in embryos having no fewer than 62 cells (Figure 1C), which we further referred to as stage V blastocysts. We further analyzed a group of embryos of a certain morphology obtained in vivo and counted the cell number after fixation (n = 19) (Figure 1D), which confirmed that the stage assessment based on embryo morphology closely correlated with cell number.

In an additional set of experiments, in vivo collected embryos were labelled with fluorescent membrane dye and immediately imaged (n = 12) (Figure 2) to visualize the shape of TE cells, which also undergoes stage-specific changes coincident with cavity expansion. During initial stages of cavitation TE cells are elongated in respect to the radial axis, some of them appearing columnar (average stage V height to width aspect ratio = 0.93), but later become progressively more flattened as the cavity expands, at first many appearing cuboidal (average stage VI height to width aspect ratio = 0.65, stage VII = 0.6), and at later stages majority appearing squamous (stage VIII ratio = 0.24) (Figure 2). Statistically significant differences in the aspect ratio were detected between all the stages except between stage VI and VII (Figure 2D).

### CDX2 is a marker of mature TE in rabbit embryos

Having established the timing of events and morphological features related to a particular stage of cavity formation and expansion, we sought to gain insight into the molecular players that are involved in TE formation in rabbits. qPCR analysis revealed that the *CDX2* transcripts are not detected in rabbit until early blastocyst (E3.25), but are found at later stages (Figure S3A).

**Figure 3:**
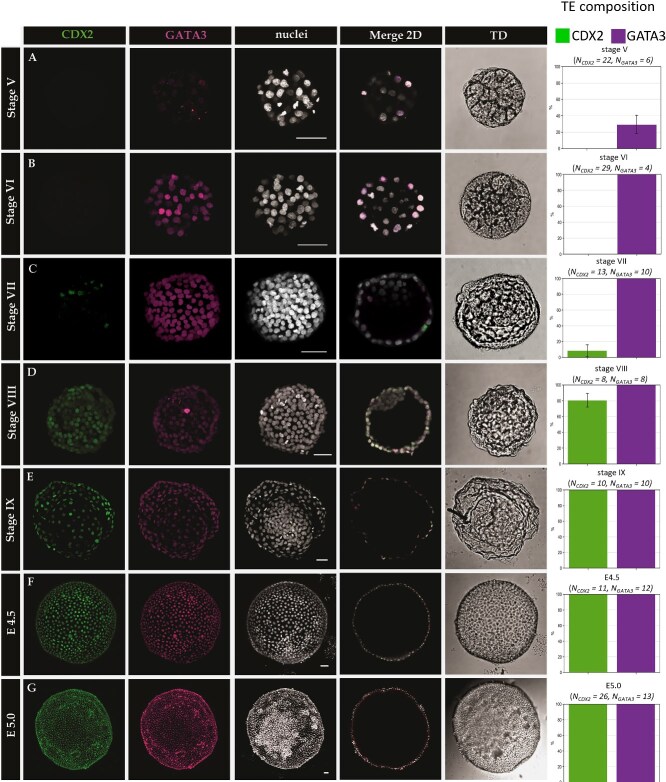
CDX2 and GATA3 are markers of TE in rabbit blastocyst. GATA3 is an early marker of TE in rabbit embryos, while CDX2 is a marker of mature TE. (A–G) Localization of transcription factors CDX2 and GATA3 at consecutive stages of rabbit embryo development. GATA3 is first detected in stage V embryos (A), appearing in the majority of outside cells. CDX2 is first detected at stage VII (C), and initially appears only in a subset of TE cells. From stage IX (E, ~E4.0), all TE cells are CDX2 and GATA3 double-positive. Each row represents a 3D reconstruction of a z-stack of a representative embryo (except merge 2D which is a single section). Green—CDX2; Magenta—GATA3; White—nuclei (Hoechst); scale bar—50 μm. Box plots represent the percentage of GATA3 and CDX2 positive cells in TE. Error bars represent standard error of the mean (SEM). Green—CDX2; Magenta—GATA3.

In agreement with the qPCR data, the immunofluorescent analysis showed no CDX2 protein in rabbit morulae (E2.0, stage IV, n = 6; stage V, n = 22), and stage V and VI early blastocyst (n = 31) (Figure 3). CDX2 was first observed in stage VII blastocysts (n = 13), specifically in the TE cells, however, it was not initially detected throughout the whole TE. Instead, it was found only in single cells scattered within the TE (on average 8.3% of CDX2–positive cells per embryo in TE, but only 23% of embryos at this stage contained CDX2-positive cells). The percentage of CDX2-positive cells in TE increased at the subsequent stages, reaching 80.6% by stage VIII (n = 8) and 100% by stage IX (n = 10), and remained localized in 100% TE cells at subsequent stages (E4.5 n = 11; E5.0 n = 26; E6.0 n = 6) (Figure 3). The observed timing of CDX2 expression suggests that it becomes upregulated alongside the process of the cavity expansion, reaching ubiquitous expression in TE cells by stage IX (~E4.0). Hence, unlike in the mouse, CDX2 is absent from morula and early blastocyst stage, and therefore not a prerequisite for initiation of TE formation and cavitation. However, in agreement with the data from human embryos [48], CDX2 in the rabbit is a marker of mature TE.

### GATA3 is an early marker of rabbit trophectoderm

As CDX2 was not detected at the early stages of cavitation, we sought the additional factors that might drive the initial stages of TE specification in the rabbit embryos. GATA3 is another important transcription factor driving the TE differentiation in the mouse [13, 14], and it has been also found in TE of other mammals, including humans [23, 26]. Therefore, we analyzed *GATA3* expression in rabbit embryos at E1.0-E6.0. The qPCR analysis revealed that *GATA3* is already expressed in E2.0 morulae, 24 hours ahead of cavitation, with the greatest transcript abundance at E3.25, the early blastocyst stage (Figure S3B). We found no GATA3 protein in stage IV rabbit morulae at E2.0 (n = 6). We further analyzed the localization of GATA3 in E2.75-E6.0 rabbit embryos (Figure 3). GATA3 was found in E2.75, fully compact late morula-stage embryos, just prior to cavitation (stage V morula) in 20% of the outside cells (n = 4). In early cavitating blastocysts, 35% of the outside cells were GATA3 positive (stage V blastocyst of around 60–70 cells) (on average 29.4% of GATA3-positive outside cells in stage V embryos irrespective of cavity presence; n = 6). At subsequent stages, GATA3 was present in all cells of the TE layer of rabbit E3.25-E6.0 blastocyst (stage VI n = 4; stage VII n = 10; stage VIII n = 8; stage IX n = 10; E4.5 n = 12; E5.0 n = 13; E6.0 n = 7) (Figure 3). By analyzing the colocalization of GATA3 and CDX2, we found that CDX2 localization is always restricted to the GATA3-positive cells (n = 24) (Figure 3). In stage VI, VII, and VIII blastocysts GATA3 was also present in a subset of ICM cells. On average 16.9% of ICM cells displayed discernible nuclear GATA3 staining, albeit much weaker than in the TE cells, with only up to three cells per ICM (located in the area adjacent to the blastocyst cavity) reaching the average fluorescence intensity of the TE cells. The average normalized ICM-to-TE GATA3 fluorescence intensity ratio for stage VI equaled 0.136, for stage VII = 0.069, and for stage VIII = 0.061 (Figure S4).

In summary, the analysis of expression and localization of CDX2 and GATA3 in rabbit embryos reveals that both factors are specific markers of rabbit TE, but exhibit differences in the timing of expression. While GATA3 is an early marker that may play a role in the initial stages of TE differentiation, CDX2 is a marker of the mature trophectoderm.

### OCT4 is expressed in both ICM and TE in the rabbit blastocyst

**Figure 4:**
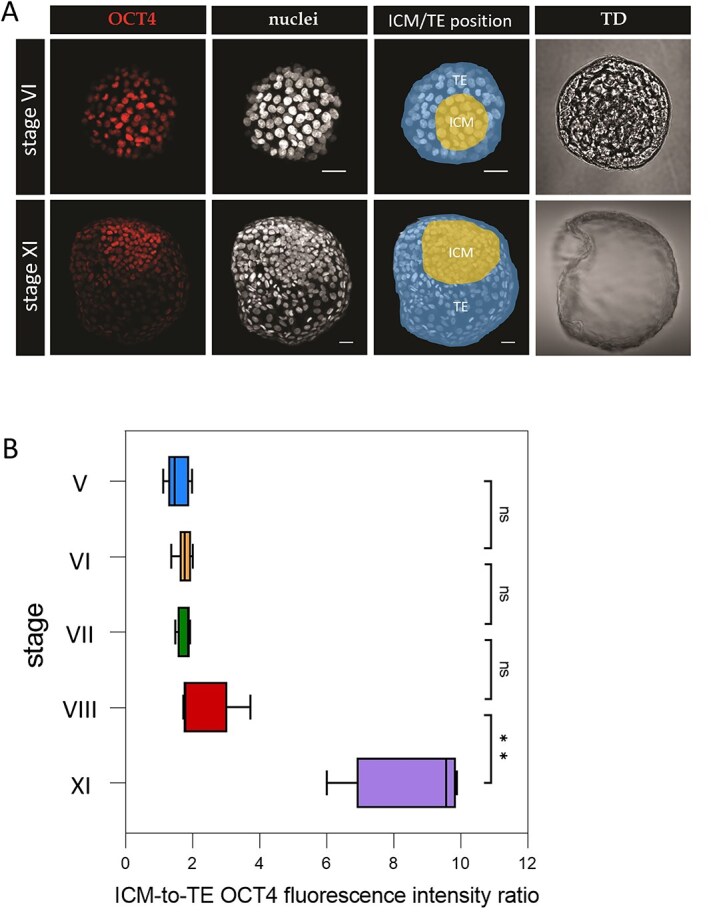
OCT4 is expressed in both TE and ICM in the rabbit blastocyst. (A) Localization of OCT4 in embryos at stage VI and stage XI. Red—OCT4; White—nuclei (Hoechst); TD—transmitted light; ICM highlighted in yellow, TE highlighted in blue. OCT4, nuclei—3D reconstruction of a z-stack, TD—single optical section. Scale bar—50 μm. (B) ICM-to-TE OCT4 fluorescence intensity ratio at stages V–VIII and XI. Stage V vs. XI (p = 0.0021), VI vs. XI (p = 0.0064), VII vs. XI (p = 0.0464), and VIII vs. XI (p = 0.0464). Kruskall–Wallis statistical test (p = 0.0094 < 0.01, ^**^ for p < 0.005). Error bars represent standard error of the mean (SEM).

Previous studies in mouse embryos suggested that CDX2 acts in ICM/TE specification by repressing the activity of octamer-binding transcription factor 4 (OCT4, encoded by the *Pou5f1/Oct4* gene), a pluripotency factor which is downregulated in mouse TE at the early blastocyst stage [12, 49]. However, it has been shown that in rabbit embryos, OCT4 persists in TE until the mid-blastocyst stage [50, 51]. To verify these findings, we analyzed *OCT4* transcript expression by qPCR in E2.0-E6.0 rabbit in vivo embryos. We found that *OCT4* was expressed at all of the stages analyzed; however, the mRNA expression significantly increased at E3.0 late morula stage (p < 0.05) and decreased in E6.0 late blastocysts (p < 0.005) (Figure S3C). We further analyzed the distribution of OCT4 protein in stage V to IX rabbit blastocysts. OCT4 protein was present in rabbit embryos at all stages analyzed and detected in nuclei of all cells in both TE and ICM (Figure 4A).

To uncover dynamics of possible OCT4 downregulation in rabbit TE, we quantified changes in ICM-to-TE fluorescence signal ratio across stages V to VIII, and additionally at stage XI (E5.0) (n = 27) (Figure 4B). At all of the analyzed stages, the normalized OCT4 fluorescence intensity was higher in the ICM than in the TE (average ratio of 1.71 for all stages analyzed), but while this ratio increased with time, there was no statistically significant difference between early and mid-blastocyst stages, from stage V to VIII (p > 0.05). However, statistically significant divergence was observed between stage XI (E5.0) and the earlier developmental stages (Figure 4B).

To summarize, both RNA and fluorescence intensity analysis suggest that in the rabbit embryos downregulation of OCT4 is not a prerequisite for TE differentiation; however, a significant decrease in OCT4 TE fluorescence intensity is observed at stage XI, suggesting further progression of TE maturation at later stages.

### Rabbit mid-blastocyst ICMs are able to restore the TE layer

As OCT4 is retained in the rabbit TE for much longer than in the mouse embryo, and its presence does not seem to be detrimental for the initial stages of TE maturation, we decided to investigate the functional consequences of such prolonged OCT4 presence in the TE. We hypothesized that isolated rabbit ICMs from later blastocyst stages might still be able to re-create the TE layer, as the high levels of OCT4 will not prevent TE differentiation.

**Figure 5:**
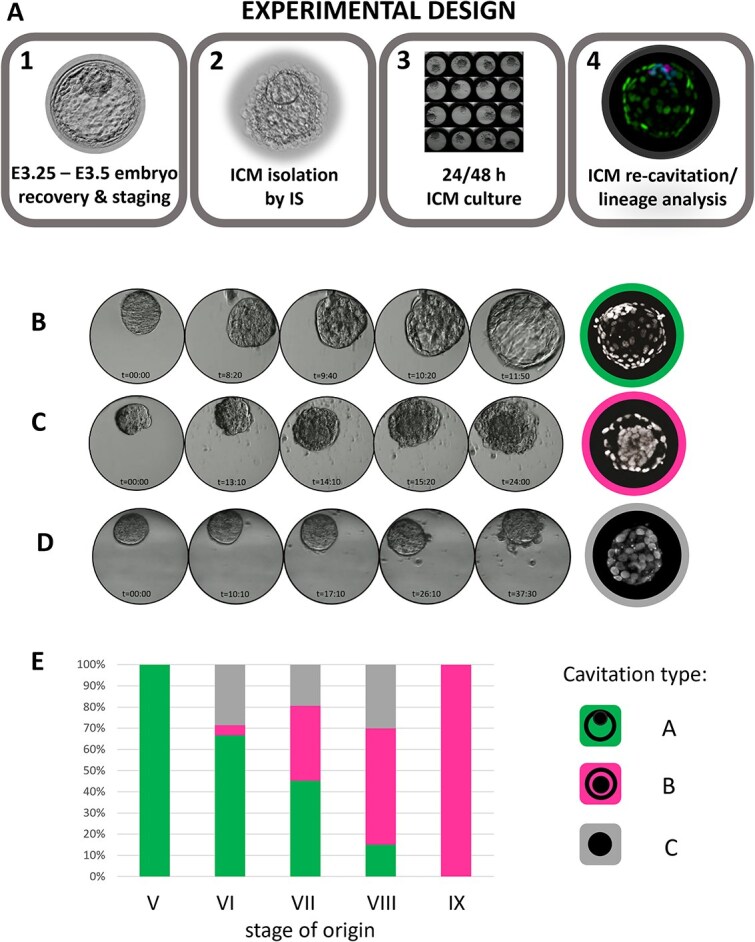
(A) Challenging ICM potency—experimental design. (1) Embryos were first selected according to the morphology and then (2) processed through immunosurgery (IS) to remove TE later. Next, (3) isolated ICMs were cultured under PrimoVision time-lapse imaging system for 24 or 48 h and then (4) fixed, immunostained for lineage-specific markers and imaged under the confocal microscope. (B–E) In vitro development of isolated rabbit ICMs. Time-lapse images of development of isolated rabbit ICMs during 48 h in vitro culture, followed by a corresponding cross-section of chromatin-stained IC-ICM (White, Hoechst). (B) Type A “blastocyst” cavitation. IC-ICMs isolated from blastocyst stages V, VI, VII follow a similar pattern of cavitation to intact rabbit embryos (compare to Figure 1). (C) Type B “halo” cavitation. IC-ICMs isolated from blastocyst stages VIII and IX fail to develop a blastocyst-like cavity but instead form a ring-shaped cavity around central cell mass. (D) Type C “no cavitation”. Despite in vitro culture, some IC-ICMs fail to re-cavitate. (E) Percentage contribution of each cavitation type in IC-ICMs after 48 h in vitro culture depending on the stage of the embryo of origin. Green—Type A, Magenta—Type B, Gray—Type C.

In order to investigate the ability of ICM to restore TE, we immunosurgically removed the TE layer from rabbit blastocysts and followed the development of the isolated ICMs cultured in vitro (further called IC-ICMs—isolated and cultured ICMs) (experimental design—Figure 5A). As we previously established that the cavitation timing does not depend directly on the time post coitum in rabbits, we staged IC-ICMs according to the geometry of the blastocyst that they were derived from, using information about geometries progression correlated with our previously developed staging system ([38]; Figure 1A). To this end, E3.25 and E3.5 blastocysts were assigned into five groups, according to the stage of rabbit blastocyst development: V, VI, VII, VIII, and IX. Isolated ICMs (n = 147) were subsequently cultured for 24 or 48 hours under PrimoVision time-lapse imaging system.

To validate the efficiency of immunosurgical TE removal, groups of embryos from different stages were labelled with fluorescent microbeads (n = 37) and imaged immediately to confirm that microbeads labelled all the outer cells of the blastocyst (TE), and none of the ICM cells. Labelled embryos were then subject to immunosurgery, and again imaged to score for the presence of labelled cells (Figure S1). This assay confirmed that no labelled cells were present following immunosurgery, and therefore immunosurgery is an efficient method of TE removal in rabbit blastocyst.

**Figure 6:**
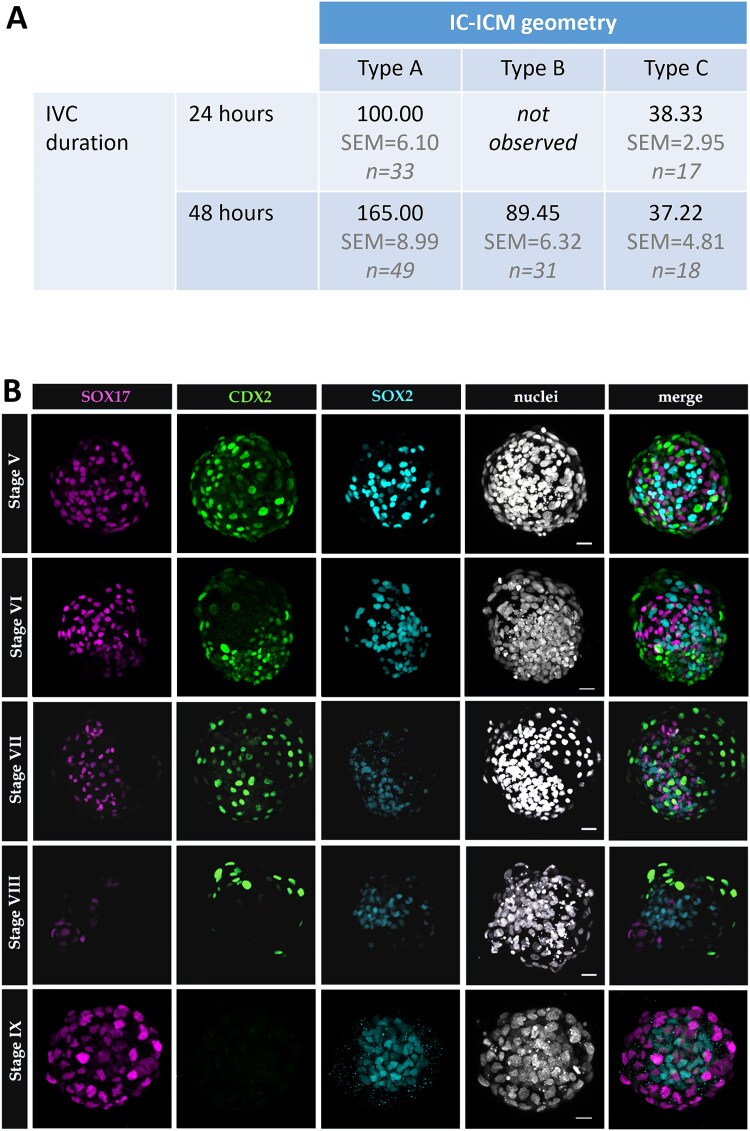
(A) Average cell numbers of IC-ICMs after 24 and 48 hours of in vitro culture, in relation to IC-ICM geometry. Differences were tested using the Kruskal–Wallis test (p = 3.6e-22 < 0.001), and subsequent pairwise comparisons were conducted using the Conover test with Holm adjustment. Statistically significant differences (p < 0.0001) were detected between all groups except 24 h type C vs 48 h type C, and 24 h type A vs 48 h type B. (B) Rabbit ICMs retain full potency until mid-blastocyst stage. Localization of PrE (SOX17), TE (CDX2) and EPI (SOX2) markers in IC-ICMs after 48 h in vitro culture. In IC-ICMs obtained from stages V and VI, CDX2 was mostly found in outside cells, while SOX2 and SOX17 were found mostly in inside cells. In IC-ICMs isolated from stage VII and VIII SOX17 is increasingly found in outside cells. IC-ICMs originating from stage IX comprise SOX17 and SOX2 positive cells, while CDX2 is not observed. Each row represents a 3D reconstruction of a z-stack of a representative embryo. Magenta—SOX17; Green—CDX2; Blue—SOX2, White—nuclei (Hoechst); scale bar—30 μm.

Following 48 h in vitro culture, we observed three distinct geometries of IC-ICMs. Type A IC-ICMs formed blastocyst-like structures, restoring the TE layer in a process similar to blastocyst cavitation in vivo, progressing through the same morphological landmarks as cavitating intact rabbit blastocysts (Figure 5B, Movie S2). Type B developed a small, halo-like cavity around a centrally localized group of cells (Figure 5C, Movie S3). Type C did not restore any cavity and remained as a compact cluster of cells (Figure 5D). ICM stage-of-origin strongly influenced IC-ICM development (Figure 5E). Stage V IC-ICMs restored type A – blastocyst-like geometry in 100% of the cases (n = 23). The potential to restore blastocyst-like structure decreased with developmental timing progression: type A geometry was observed in 66.7% of stage VI IC-ICMs, 45.2% of stage VII, 15% of stage VIII and no type A geometry was re-formed from stage IX IC-ICMs. Concomitantly, halo-like cavity (type B) was not observed in IC-ICMs from stage V, but it was found with an increasing incidence at later stages – 4.8% in stage VI, 35.5% in stage VII, 55.5% in stage VIII, and 100% IC-ICMs from stage IX restored halo-like cavity (n = 8). Cell number of IC-ICMs was also correlated with specific geometries (Figure 6A)– after 24 h of culture, type A IC-ICMs contained on average 100 cells, while non-cavitated IC-ICMs – 38.33 cells. After an additional 24 h of culture (48 h total) cell numbers increased to 165.00 for type A and 89.45 for type B, and remained constant for type C (37.22).

### CDX2 and GATA3 expression coincides with the restoration of functional TE-layer in recavitated IC-ICMs

**Figure 7:**
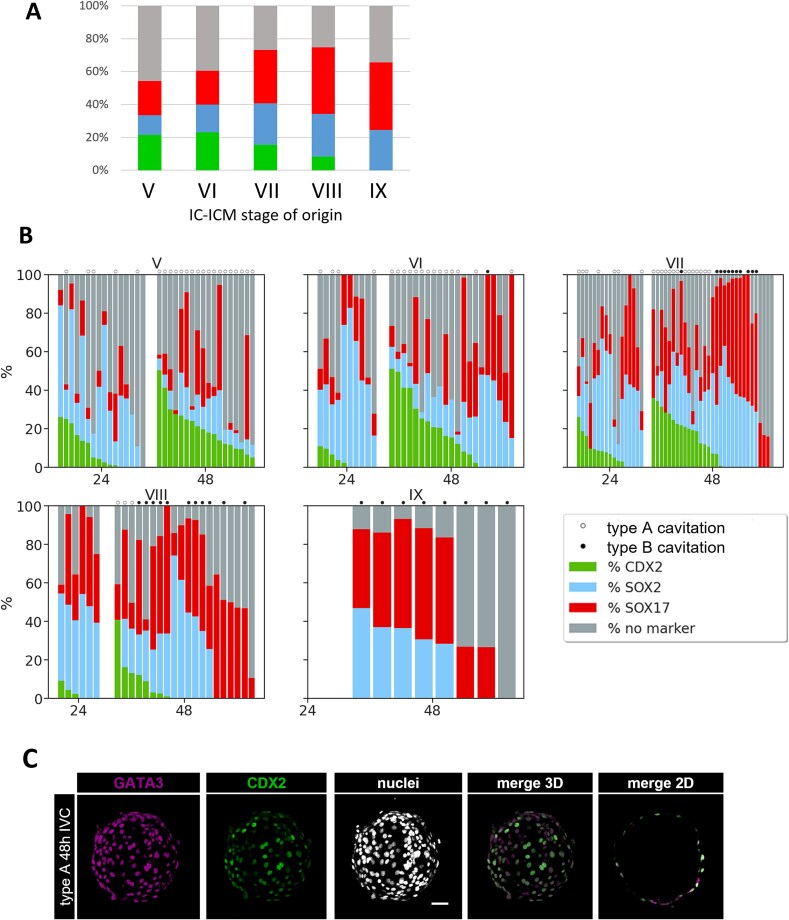
Contribution of CDX2, SOX2, SOX17 positive, and triple-negative cells in IC-ICMs isolated from stage V, VI, VII, VIII, and IX blastocysts, after 24 and 48 h of in vitro culture. (A) Average contribution of each cell type in IC-ICMs after 48 h of in vitro culture. Green—CDX2; Blue—SOX2; Red—SOX17; Gray—triple-negative cells. (B) Contribution of each cell type in individual IC-ICMs after 24 and 48 h of in vitro culture. Each column represents a single IC-ICM, data are presented in order of the decreasing CDX2 contribution. IC-ICMs with type A cavitation are marked with “o”, type B cavitation – with “•”. (C) Restored TE of IC-ICMs expresses TE markers GATA3 and CDX2. Localization of GATA3 and CDX2 in IC-ICMs after 48 h of in vitro culture. In type A IC-ICMs GATA3 is present in all cells of the newly reformed TE, and CDX2 is found in the majority of those cells. 3D reconstruction of a z-stack of a representative embryo, and merge 2D of a single section. Magenta—GATA3; Green—CDX2; White—nuclei (Hoechst); scale bar—50 μm.

IC-ICMs (n = 98) were fixed and stained for lineage markers: SOX2 for Epi, SOX17 for PrE and CDX2 for TE (Figure 6B). We noticed that the number of CDX2^+^ cells decreases with the stage of isolated IC-ICM, i.e., IC-ICMs isolated from later stages (VII, VIII) have fewer CDX2^+^ cells (fewer recavitated IC-ICMs with CDX2^+^ cells and also fewer CDX2^+^ cells per each recavitated IC-ICM). In ICMs isolated from stage IX, we did not find any CDX2^+^ cells, suggesting that at this stage, all ICM cells lost their ability to differentiate towards TE (Figure 7A, B).

In the rabbit blastocyst, GATA3 is an early TE marker that precedes CDX2 expression (Figure 3). To further uncover the extent of TE specification in recavitated IC-ICMs, we additionally analyzed GATA3 distribution. After 48 h of culture, GATA3 was present in the outside cells of 70% of recavitated IC-ICMs (n = 20) originating from V-IX stage blastocysts. Re-formed TE of type A IC-ICMs contained mostly CDX2^+^ GATA3^+^ double-positive cells, although in 14.2% of embryos we also found GATA3^+^, CDX2^−^ cells (n = 14), which resembles the expression pattern of early in vivo blastocyst (Figure 7C). Similarly to intact embryos, we found no CDX2^+^ GATA3^−^ cells in any of the IC-ICMs.

### TE specification is initiated in the rabbit ICM shortly after isolation

To better understand the dynamics of IC-ICM development and differentiation, we additionally analyzed stage V-VIII IC-ICMs after a shorter, 24-h in vitro culture (n = 49) (Figure 7). We found that IC-ICMs isolated from stage V restored type A cavity in 31.3% of the cases, from stage VI in 40% and from stage VII in 41% of the cases, but no cavity was formed after 24 h culture of IC-ICMs from stage VIII. This indicates that TE re-formation is a relatively rapid process, as we observed type A cavitation 24 h after ICM isolation, and in some cases (in time-lapse recordings) as early as within 10 h of in vitro culture. In contrast, type B cavitation was never observed after 24 hours of IC-ICM in vitro culture.

### IC-ICMs express markers of TE, Epi, and PrE lineages

**Figure 8:**
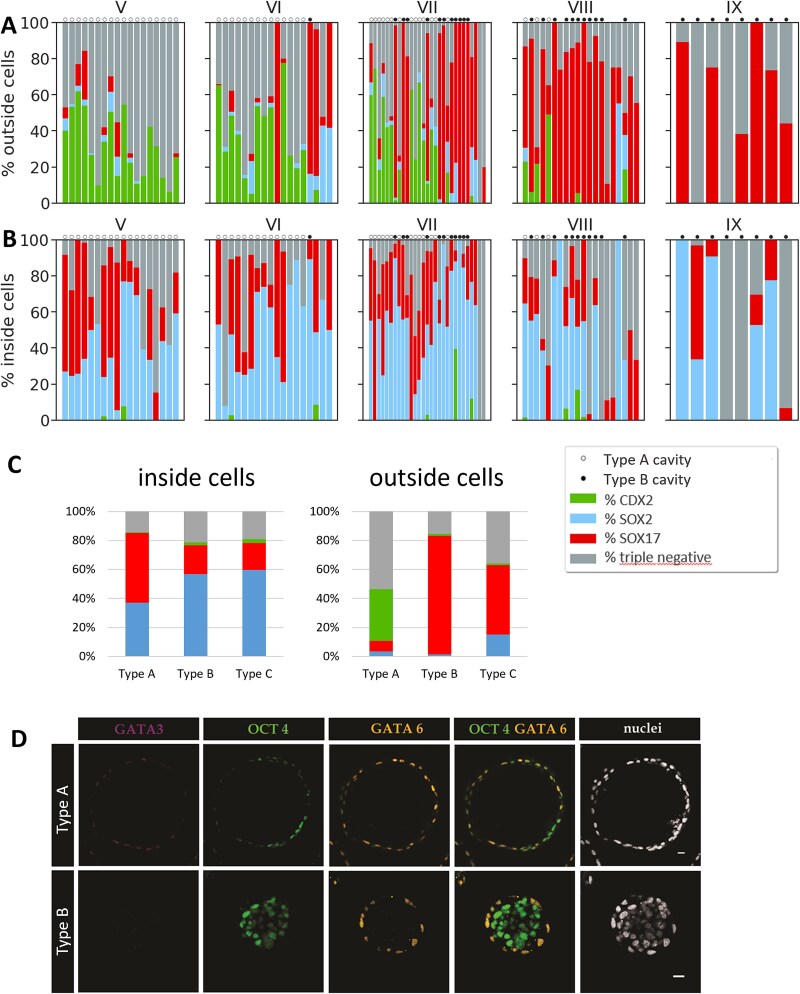
Percentage contribution and differentiation of inside and outside cells in all IC-ICMs isolated from stage V, VI, VII, VIII, and IX blastocysts after 24 and 48 h in vitro culture. (A) CDX2, SOX2, SOX17— positive, and triple-negative cells in IC-ICMs in outside compartment. (B) CDX2, SOX2, SOX17— positive, and triple-negative cells in IC-ICMs in inside compartment. IC-ICMs with type A cavitation are marked with “o”, type B cavitation – with “•”. Each column represents a single IC-ICM, data are presented in order of the decreasing cell count in the IC-ICM. (C) Averaged composition of inside and outside compartment of type A vs type B and type C IC-ICMs, irrespective of the stage of origin. Data replotted from Figure 7. (D) IC-ICMs express early lineage-specific markers. Localization of GATA3, OCT4, and GATA6 in IC-ICMs after 48 h of in vitro culture. In type A IC-ICMs (represented here by one originating from stage VI), GATA3, GATA6, and low levels of OCT4 are observed in outside cells, and higher levels of OCT4 in inside cells. In type B IC-ICMs (represented here by one originating from stage VIII) GATA6 is observed in outside cells and OCT4- in inside cells. Each row represents a single section of a z-stack of a representative embryo. Magenta—GATA3; Green—OCT4; Yellow—GATA6; White—nuclei (Hoechst); scale bar—50 μm.

Having established that isolated rabbit ICMs are able to restore a blastocyst-like structure, which has an outside GATA3^+^, CDX2^+^ TE layer, we asked whether the inner cells of the IC-ICM also differentiate into appropriate lineages. In order to do this, we tested IC-ICMs for the presence of PrE (SOX17) and Epi (SOX2) lineage markers (Figures 6 and 7). The majority of IC-ICMs contained both SOX2^+^ and SOX17^+^ cells, and double-positive cells were never present. Epiblast marker SOX2 was present in 86.5% of all recavitated IC-ICMs (n = 96). The percentage of SOX2^+^ cells increased in IC-ICMs derived from later stages – namely, IC-ICMs from stage V contained on average 12.3% of SOX2^+^ cells, from stage VI – 16.9%, from stage VII – 23.9%, from stage VIII – 22.4% and from stage IX – 25.2%. In all of these cases, SOX2^+^ cells were present in the inside compartment, and in 39.6% of IC-ICMs a small number of SOX2^+^ cells was also found in the outside compartment (1–5 cells per embryo) (Figure 8A, B). PrE marker SOX17 was present in 89% of recavitated IC-ICMs (n = 96). The overall percentage of SOX17^+^ cells was higher in the IC-ICMs derived from later stages – namely, IC-ICMs from stage V contained 22% SOX17^+^ cells, from stage VI – 22%, from stage VII – 32%, from stage VIII – 40.4% and from stage IX – 42.7%. In 84.4% of IC-ICMs SOX17 was found in the inside cells, and in 57.3% in the outside cells (Figure 8).

To further characterize the triple-negative cells which lacked either of the late lineage markers, we additionally analyzed the localization of early lineage markers – GATA3 (TE), GATA6 (PrE) and OCT4 (Epi) (n = 7, Figure 8D). Our analysis revealed that all of the outside cells in the type A IC-ICMs (restored TE cells), including those negative for the previously analyzed late lineage markers, were positive for all of these markers, while outside cells in type B IC-ICMs were positive for GATA6 but not OCT4 or GATA3, and inside cells in type B IC-ICMs were positive for OCT4, but not for the extraembryonic lineage markers.

### Rabbit IC-ICMs isolated from late blastocyst form SOX17^+^ outside layer

Next, we analyzed whether each of the observed cavitation geometries (type A, B or C) is correlated with a specific expression pattern of lineage-specific transcription factors in IC-ICMs following 48-hour in vitro culture (Figures 7 and 8).

Analyzing the distribution of the TE, PrE, and Epi markers in the IC-ICMs after 48 h culture we noticed that CDX2 and SOX17-positive cells were present in both inside and outside locations. We noted that the contribution of SOX17^+^ cells to the outside compartment was higher in the IC-ICMs originating from later-stage rabbit blastocysts. SOX17^+^ cells constituted only 5.3% of outside cells in IC-ICMs derived from stage V, 12% from stage VI, 28.1% from stage VII, 55.5% from stage VIII and 62.8% from stage IX. Overall, in 41% of IC-ICMs SOX17^+^ cells were present in the outside layer only. To quantify that further, we analyzed the distribution of cells within the IC-ICMs in the inside versus outside compartments after 48 hours of culture in relation to the type of cavity restoration geometry (Figure 8B).

CDX2-positive cells were present in nearly all of the type A IC-ICMs from all stages (48 out of 49 IC-ICMs), and in all of these cases CDX2 was predominantly present in the outside cells. Only 2 IC-ICMs from stage V, 1 IC-ICM from stage VI and 1 from stage VIII contained 1–3 CDX2-positive cells in the inside compartment (Figure 8A, B).

The blastocyst-like structures (type A) contained all three cell lineages, properly sorted into their respective compartments (Figures 6B, 8C). In addition to CDX2 and GATA3 positive outside TE layer, type A IC-ICMs re-formed an ICM which contained SOX2^+^ cells (in 91% of the cases) and SOX17^+^ cells (85% of the cases) (Figure 8B). In none of the cases the analyzed markers were colocalized, suggesting that rabbit IC-ICMs are capable of proper differentiation and segregation into the first three cell lineages within 48 h period.

In type B IC-ICMs after 48 h culture, the percentage of CDX2-positive cells almost never exceeded 15% per ICM (in 30 out of 31 IC-ICMs). No CDX2-positive cells were present in type B IC-ICMs from stages VI and IX (Figure 7), whereas 45% of stage VII and 18% of stage VIII IC-ICMs contained some CDX2-positive cells, mostly located in the inside compartment. The outside layer of type B IC-ICMs was largely devoid of CDX2 (no CDX2-positive cells in 26 out of 31 type B IC-ICMs, and only up to five cells in the remaining 5).

Careful analysis of the composition of the outside layer in relation to the type of cavitation geometry revealed that while in 95.9% of type A IC-ICMs outside layer contained CDX2^+^ cells, in 93.6% of type B IC-ICMs the outside layer was composed mostly of SOX17^+^ cells (Figure 8A).

To further understand the correlation between inside and outside compartment composition and re-cavitation potential, we analyzed the lineage composition in IC-ICM embryos classified according to the IC-ICM geometry (Figure 8C). Our analysis revealed that the inside compartment almost always contained both SOX2-positive and SOX17-positive cells, irrespective of the cavitation type. CDX2-positive cells were also occasionally present, but on average constituted less than 4% of the compartment. The composition of the outside compartment was strongly correlated with IC-ICM geometry and hence the ability of ICM to restore TE layer. Type A IC-ICMs, which contained on average 35.5% CDX2-positive cells and less than 10% of ICM lineages (SOX2-positive and SOX17-positive cells, jointly), were able to successfully restore the TE (type A). However, in those IC-ICMs in which CDX2-positive cells constituted on average less than 1.5% of the outside compartment, TE reconstitution was never observed (type B and type C). Additionally, while the high (81.77%) contribution of SOX17-positive cells in the outside compartment correlated with type B IC-ICM, the lower contribution of SOX17^+^ cells (47.93%) combined with SOX2^+^ (15%) present in the outside compartment correlated with failure to cavitate (type C).

Our data indicate that rabbit ICMs from earlier stages have a higher capability to restore the TE layer. This ability is gradually lost, although it is still maintained in some ICMs from stage VIII. Later-stage IC-ICMs form an outside layer of SOX17-positive cells and are only able to restore the halo-like cavity. In summary, the ability to differentiate towards TE, defined by both type A blastocyst-like cavity re-formation and expression of CDX2 and GATA3 in newly formed TE, is gradually lost, giving way to the formation of SOX17-positive outside layer and type B “halo” cavity geometry.

## Discussion

Recent studies suggest the existence of substantial differences in the formation of the first cell lineages between mammals (reviewed in [8]). However, the significance and the origin of these differences are not yet fully understood. In this work, we investigate the TE differentiation and temporal link between the cellular plasticity of the ICM cells and the cell lineage specification process using rabbit isolated ICMs.

In the rabbit embryo, the morphological distinction between the TE and the ICM becomes first apparent around E3.0, in embryos composed of around 64 cells. Our observation of in vivo obtained embryos, as well as time-lapse imaging of embryos collected at the late morula stage and subsequently cultured in vitro, reveals distinct features of early rabbit blastocyst morphology and cavity expansion. Indeed, our observation suggests several differences between the cavity formation of mouse versus rabbit embryos. In rabbit embryos, initiation of cavity formation appears in a form of a U-shaped slit between the outer layer of the morula and its inner cells. Unlike in the mouse embryos [52], cells of this outer layer (i.e., trophectoderm) are not flattened or stretched, but maintain the cuboidal shape, similarly to early bovine TE cells [53].

CDX2 and GATA3 are the main drivers of TE fate in the mouse [12–14]. Their TE-specific localization has also been confirmed in a number of other mammalian species (reviewed in [8]). Previous studies suggested a late onset of CDX2 expression in rabbit embryos [17], as CDX2-positive cells were found only in a subset of E4.0 early blastocyst TE. In agreement with this study, we found no CDX2-positive cells in E3.0 morulae prior to cavitation. Moreover, a more detailed analysis of rabbit embryos between E3.0 and E4.0 allowed us to establish that CDX2 becomes first apparent in stage VII blastocyst. Interestingly, CDX2 can be initially detected only in single cells and becomes expressed ubiquitously throughout TE only at stage IX (~E4.0). This analysis shows that cavitation in rabbit embryos is executed in the absence of CDX2, and therefore *CDX2* expression is not a prerequisite for the initial stages of TE formation. A similar observation has been made in pig and human embryos, where the initiation of the TE formation takes place before the CDX2 becomes expressed [16, 48, 54]. Indeed, in bovine and porcine embryos, as well as in the mouse, downregulation of *CDX2* does not disrupt cavitation and early stages of TE formation [12, 15, 18, 21, 55]. However, CDX2 is necessary for the later stages of TE differentiation, as loss of CDX2 results in failure in correct TE proliferation (cow [15]), maintenance of epithelial integrity (mouse [12, 55]) and cell polarity (pig [16]) which ultimately leads to failure in embryo implantation. Despite the lack of a clear requirement of early *Cdx2* expression for TE cell fate specification, it is expressed in mouse morulae as early as the 8-cell stage [12, 56]. This disparity may be explained by possible differential regulation of pre- and post-cavitation *Cdx2* expression. While the latter is dependent on the Hippo pathway [7, 57–60], the early, morula stage expression is regulated by Notch signaling in the mouse [61, 62] and might be entirely missing in the other mammalian species.

In search of a factor capable of initiation of the TE program, and a suitable early marker of the TE lineage, we investigated the distribution of GATA3, a transcription factor previously reported in mouse [13, 14], which has been also recently shown to be a TE- specific marker in bovine and human embryos [23, 26]. Detailed immunofluorescent analysis of rabbit preimplantation embryos showed that GATA3 is an early, specific and robust marker of rabbit TE (Figure 3), in agreement with a recent transcriptomic study [63]. It is first detected in the outside cells of rabbit stage V late compact morula (at ~E3.0), and throughout subsequent development, it is present specifically in all cells of the rabbit TE. The co-localization analysis also showed that CDX2 protein expression is restricted only to GATA3-positive cells (although up to stage VIII, GATA3-positive, CDX2-negative cells are present), which suggests that GATA3 might be a prerequisite for CDX2 expression. Additionally, we detected *GATA3* transcripts 24 h prior to protein, which may suggest that the TE program is initiated in the rabbit embryo as early as E2.0, or that it might require an additional trigger just before cavitation.

Analysis of *OCT4* mRNA expression in rabbit embryos in vivo revealed a significant increase in transcript levels at E3.0 late morula stage. Consecutively, at E3.25 early blastocyst stage, OCT4 became downregulated (p < 0.05), while *GATA3* transcript levels significantly increased (p < 0.05). However, at this stage, we have not detected *CDX2* transcripts. Analysis of OCT4 protein localization in in vivo rabbit blastocyst confirmed its presence in both ICM and TE, in agreement with previous reports [50, 51]. This indicates that in rabbit, OCT4 downregulation is not necessary to initiate or maintain the TE program up to the mid-blastocyst stage, and further supports inter-species differences in gene regulatory networks, underlying differences in the mechanisms of lineage specification. Indeed, OCT4 has been detected in both ICM and TE in a number of species, including human, cattle and pig [15, 64]. Although in the mouse embryo CDX2 acts as a direct transcriptional repressor of *Oct4* [65, 66], it is not the case in porcine embryos [67], where CDX2 has been shown to promote OCT4 proteasomal degradation, but not transcriptional control. Comparative studies of bovine and mouse embryos revealed the existence of a cis-acting regulatory region required to suppress TE-specific *Oct4* transcription in murine embryos [15]. This species-specific enhancer has been found in mice, but not in cattle, humans, and rabbits [15], clearly confirming differences in gene regulatory networks governing early lineage specification between mammals.

In the mouse embryo, both TE cells and ICM cells become mostly restricted in their developmental potential soon after cavitation [31–33, 68]; however, isolated cells of morphologically distinguishable TE are able to contribute to ICM lineages in chimaera studies of bovine and human embryos [15, 69]. Recent studies have also shown that in bovine embryos, ICM potential is maintained for a longer period, as isolated ICMs of 6.0 dpf blastocysts are able to regenerate the TE layer [36]. This raises an interesting question of inter-specific differences in the ICM/TE differentiation mechanism. An early restriction of cell fate in mouse embryos might be a direct result of unusually rapid preimplantation development of the mouse, which is ready for implantation in just 4 days. In other mammalian species, the preimplantation period is extended over several days or even weeks, which may not require such immediate specification decisions (reviewed in [70]), and may result in a longer period of plasticity or even totipotency.

**Figure 9:**
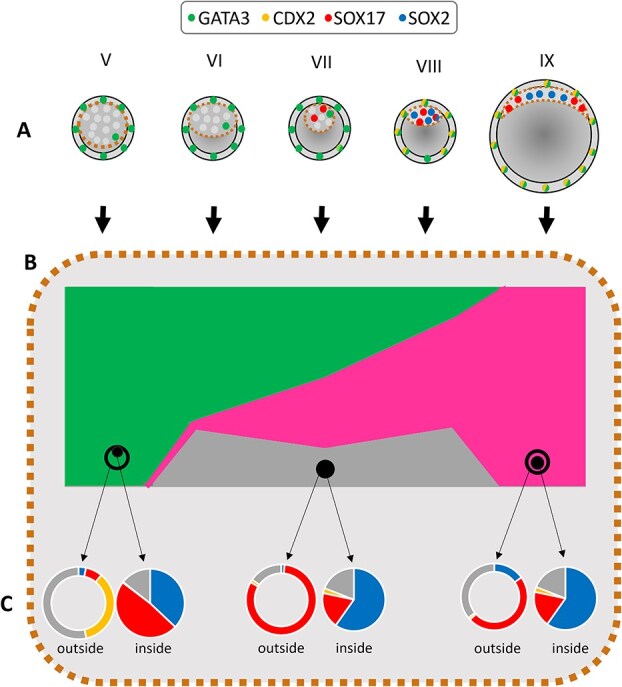
Model of relationship between lineage specification and cell plasticity in rabbit blastocyst. (A) In rabbit embryos, GATA3 is already present in the trophectoderm of stage V and VI early blastocyst, while CDX2 becomes upregulated at stages VII–VIII, and is subsequently coexpressed with GATA3 in all TE cells. ICM cells start upregulating PrE marker SOX17, and at stage VIII ICM is mostly (and by stage IX—exclusively) composed of SOX2— and SOX17—positive cells, suggesting that at that stage, ICM lineage specification is predominantly completed. Green—GATA3; Yellow—CDX2; Blue—SOX2; Red—SOX17; Gray—triple-negative cells. (B) ICMs of early rabbit blastocyst have the ability to re-form blastocyst-like structures (Green, type A), which is progressively lost in a stage-sensitive manner, giving way to the formation of secondary halo-like cavity (Magenta, type B). Some of the isolated ICMs fail to form any type of cavity, within the frame of 48 h IVC, but form SOX17-positive outer layer, suggesting the existence of transient state (Gray, type C), similar in differentiation potential to type B (Magenta), but lacking cavity. ICMs of early rabbit blastocyst (stages V–VII) which are able to form blastocyst-like structures (type A), have unrestricted lineage specification potential, evidenced by lineage specification of EPI (SOX2), PrE (SOX17), and TE (CDX2) lineages. This ability is progressively lost, corresponding to higher levels of EPI and PrE-lineage specification in ICM, and halo-like structures (type B) emerging from later-stage ICMs form predominantly SOX17- positive outer layer. Pie charts represent averaged lineage composition of inside and outside compartment of types A, B, and C IC-ICMs (replotted from Figure 8C). Yellow—CDX2; Blue—SOX2; Red—SOX17; Gray—triple-negative cells.

Our analysis of the development of isolated rabbit ICMs uncovered that after 48 h culture, they were able to form two distinct types of cavitated structures. Type A had a typical morphology of an unperturbed blastocyst, with a single large cavity and ICM located peripherally within it. Type B formed a halo-like cavity around a centrally located mass of cells. We also observed that the morphological changes during type A cavity re-formation progressed through the same stages as in normal blastocyst development (with respect to the order of events, shape of the cavity and shape of the outer layer of cells). IC-ICMs potential to form each type of cavity depended on the stage the ICM was derived from, with earlier stages (V, VI) preferentially forming type A, and later stages (VIII, IX) preferentially forming type B cavity (Figure 5E, Figure 9). Moreover, we noted that type A cavitation is a relatively more rapid process, as after 24 h of in vitro culture we have already observed type A, but no type B morphology, further supporting different mechanism of cavitation. Immunofluorescent analysis revealed that type A structures contained cells expressing markers of PrE (SOX17), Epi (SOX2) and TE (CDX2 and GATA3) localized exclusively in the appropriate compartment. In addition to correctly specified SOX2^+^, SOX17^+^ and CDX2^+^ cells, IC-ICMs also contained varying amounts of triple-negative cells. Despite lacking these late lineage markers, those cells were positive for early lineage markers- OCT4, GATA6 and GATA3 (Figure 8D). Such cells are present in in vivo early-to-mid blastocyst stage embryo but are absent from expanded blastocyst where the lineage specification has been largely completed (from E4.0; [45] and this manuscript; Figure S5). This allows us to conclude that stage V and VI (and to some extent stage VII) ICMs are not restricted to EPI or PrE cell fate, but retain the potential to differentiate into all three lineages of the mammalian blastocyst, including the TE.

The ability for type A cavity regeneration is progressively diminished in the ICMs isolated from later stages and entirely lost by stage IX (~E4.0 expanded blastocyst). Moreover, we have noted that in type B IC-ICMs, the outer layer is composed of CDX2-negative, SOX17-positive cells. Our previous studies showed that stage IX ICM is entirely composed of already sorted EPI and PrE compartments [45], therefore this loss of plasticity to regenerate TE seems to coincide with differentiation of the entirety of ICM cells to PrE and Epi lineages (Figure 9, Figure S5C). Mouse isolated ICMs lose the re-cavitation potential already at the early blastocyst stage [31, 32], but similarly to rabbit embryos, later ICMs form an outer layer of PrE, GATA4-positive cells, even though a halo-like cavity was not observed in this case [43]. A recent analysis of human ICM explants revealed their potency to generate TE cells in vitro, but this ability was later lost to allow for amnion cells differentiation [26].

In summary, this body of evidence suggests that the TE specification program potentially employs the same set of core lineage-specific transcription factors, GATA3 and CDX2, across the eutherian mammals, although more functional studies in different mammalian species are needed to fully elucidate this process. Importantly, loss of developmental plasticity and disparity of timing in ICM versus TE differentiation reflect inter-species differences, coincident with variances in species anatomy and reproductive physiology. The precise length of the TE-permissive period varies between species and likely depends on the length of the preimplantation period (4 days in mouse to over 20 days in cow) [71, 72], and more specifically on the timing of differentiation of other extraembryonic lineages. Taken together with recent findings in other species, our data indicate that mammalian ICM cells have a time-limited potential to regenerate TE, but this ability has to be gradually lost to allow for differentiation into another extraembryonic epithelial layer.

## Supplementary Material

Fig_S1_ioaf157

Fig_S2_ioaf157

Fig_S3_ioaf157

Fig_S4_ioaf157

Fig_S5_ioaf157

Movie_S1_ioaf157

Movie_S2_ioaf157

Movie_S3_ioaf157

Fig_2_resub_ioaf157

Supplementary_Figure_caption_ioaf157

Supplementary_Movie_caption_ioaf157

## Data Availability

The data underlying this article are available in the article and its online supplementary material.
