# Supplementary figures and images for: Stage-sensitive potential of isolated rabbit ICM to differentiate into extraembryonic lineages
Source: Biol Reprod. 2025 Jul 22;113(5):1102–20. doi: 10.1093/biolre/ioaf157 (PMC12621310; doi:10.1093/biolre/ioaf157)

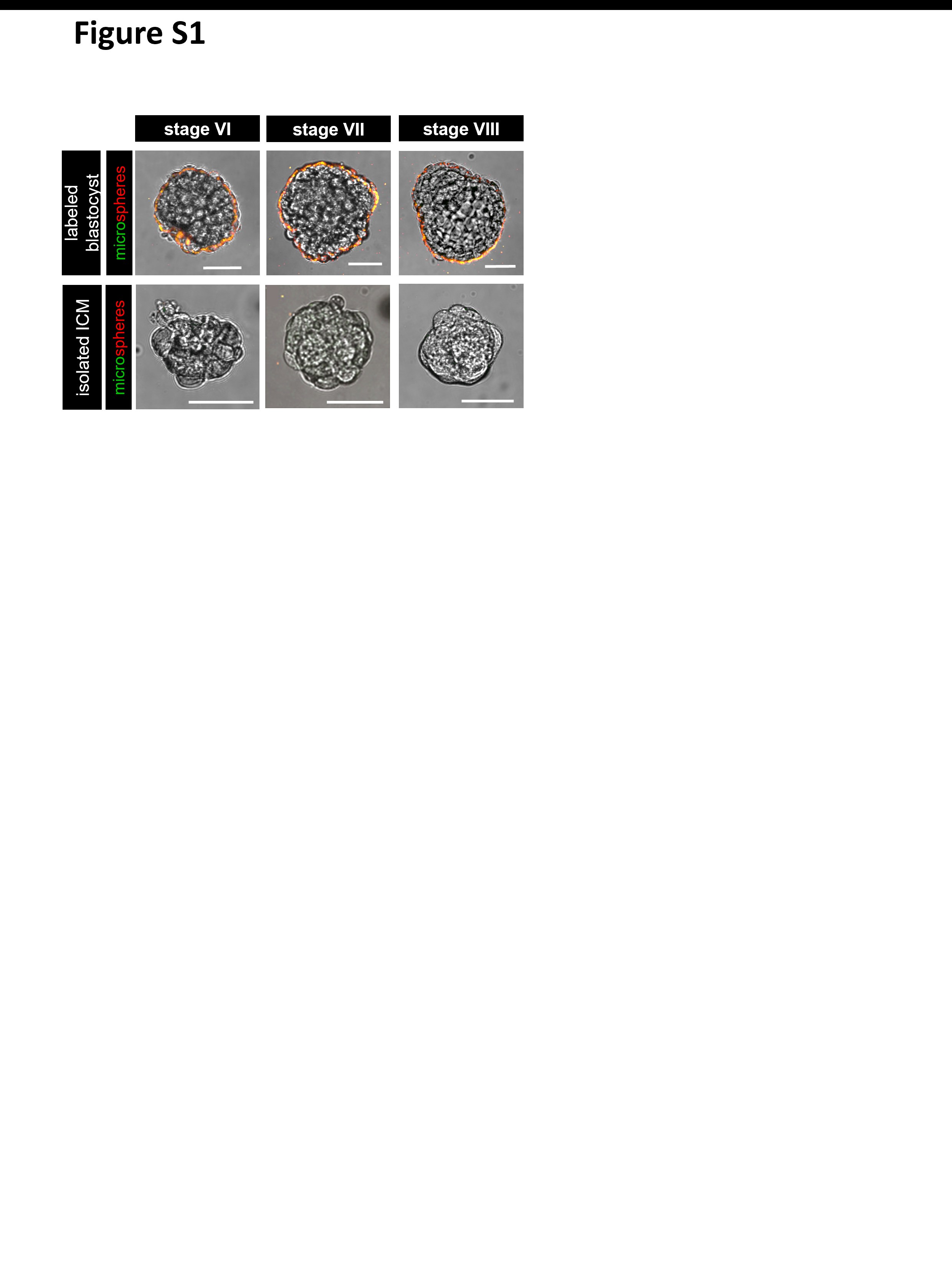

Supplement: Fig_S1_ioaf157 [file fig_s1_ioaf157.jpeg]

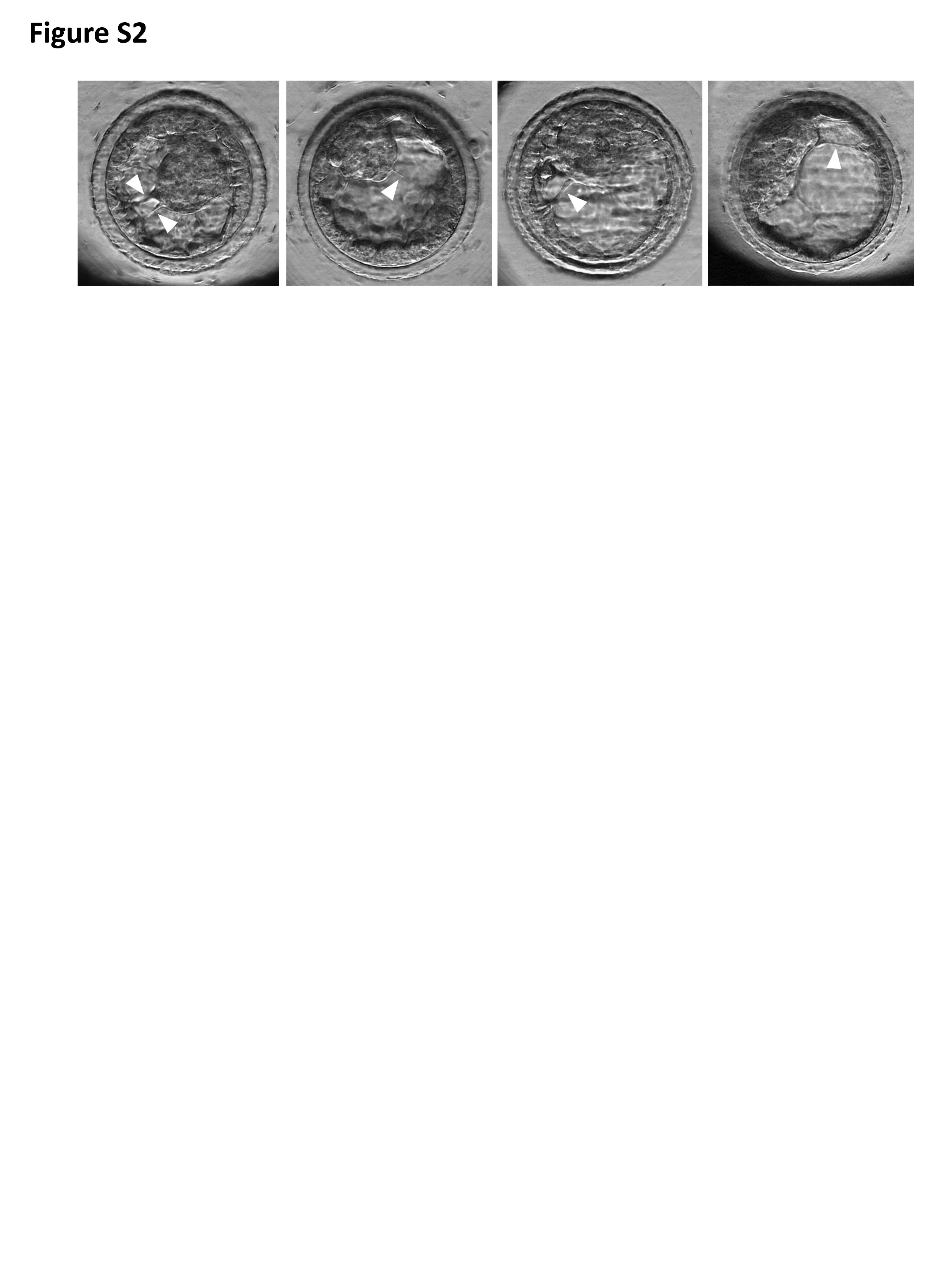

Supplement: Fig_S2_ioaf157 [file fig_s2_ioaf157.jpeg]

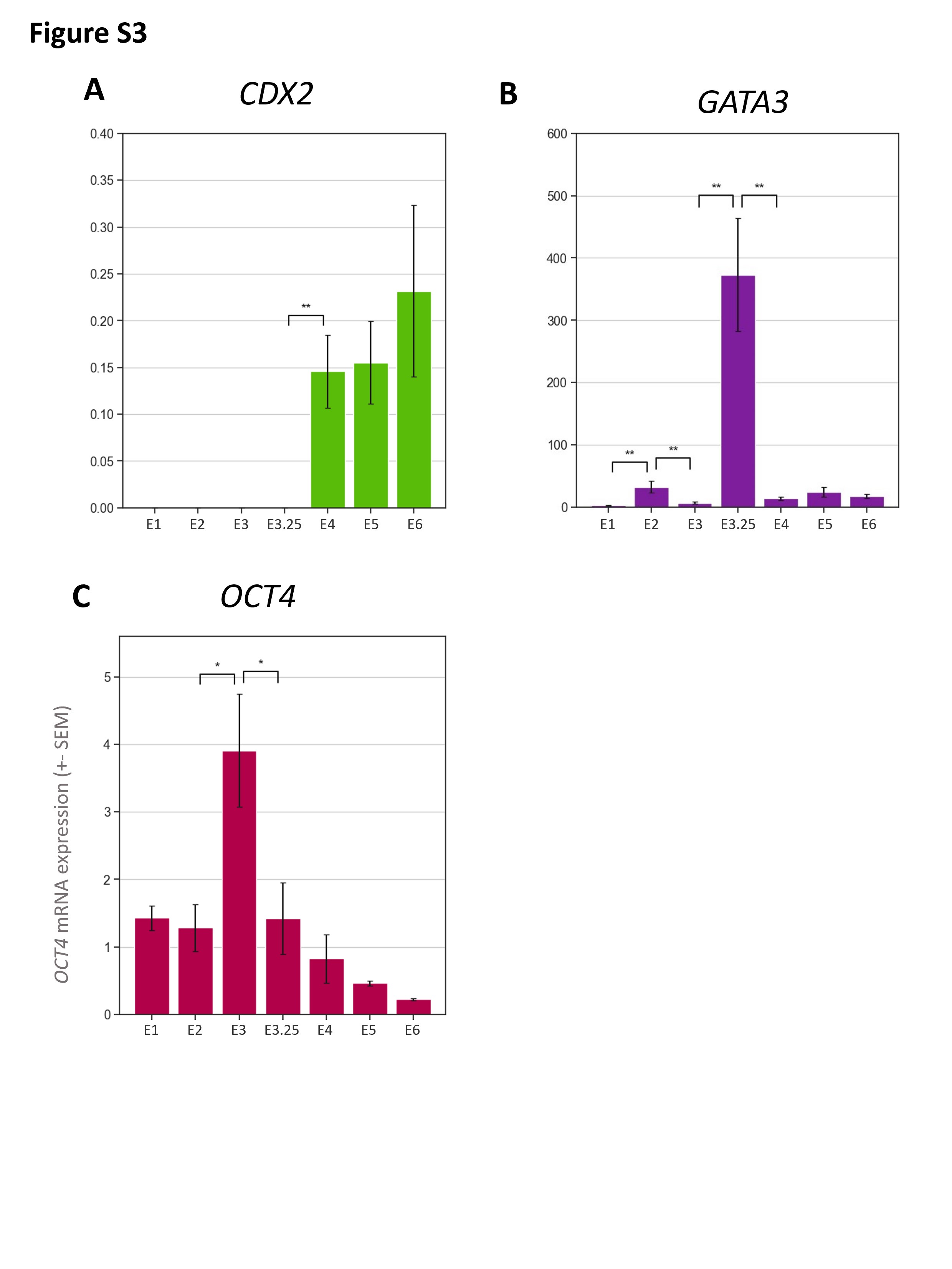

Supplement: Fig_S3_ioaf157 [file fig_s3_ioaf157.jpeg]

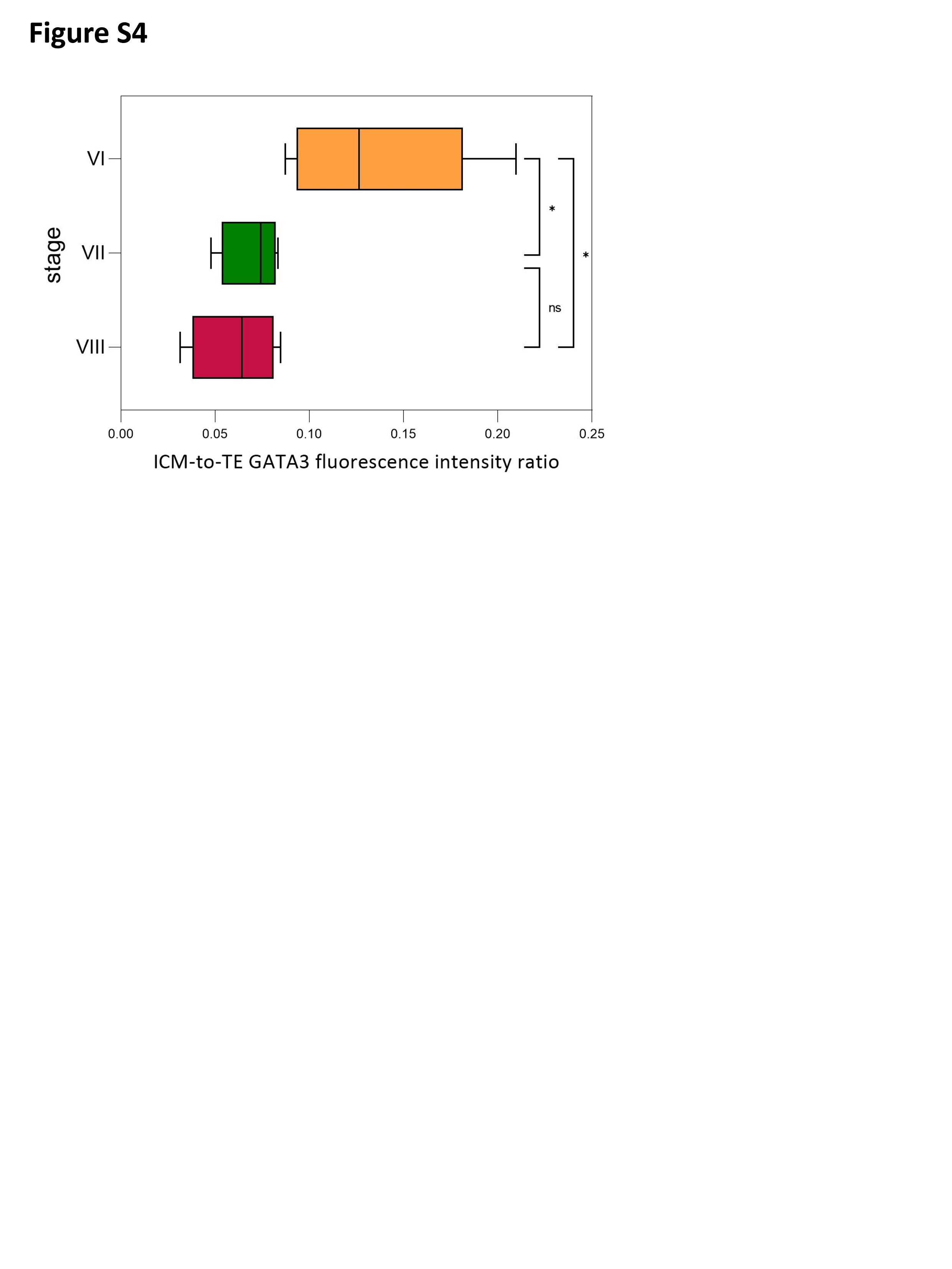

Supplement: Fig_S4_ioaf157 [file fig_s4_ioaf157.jpeg]

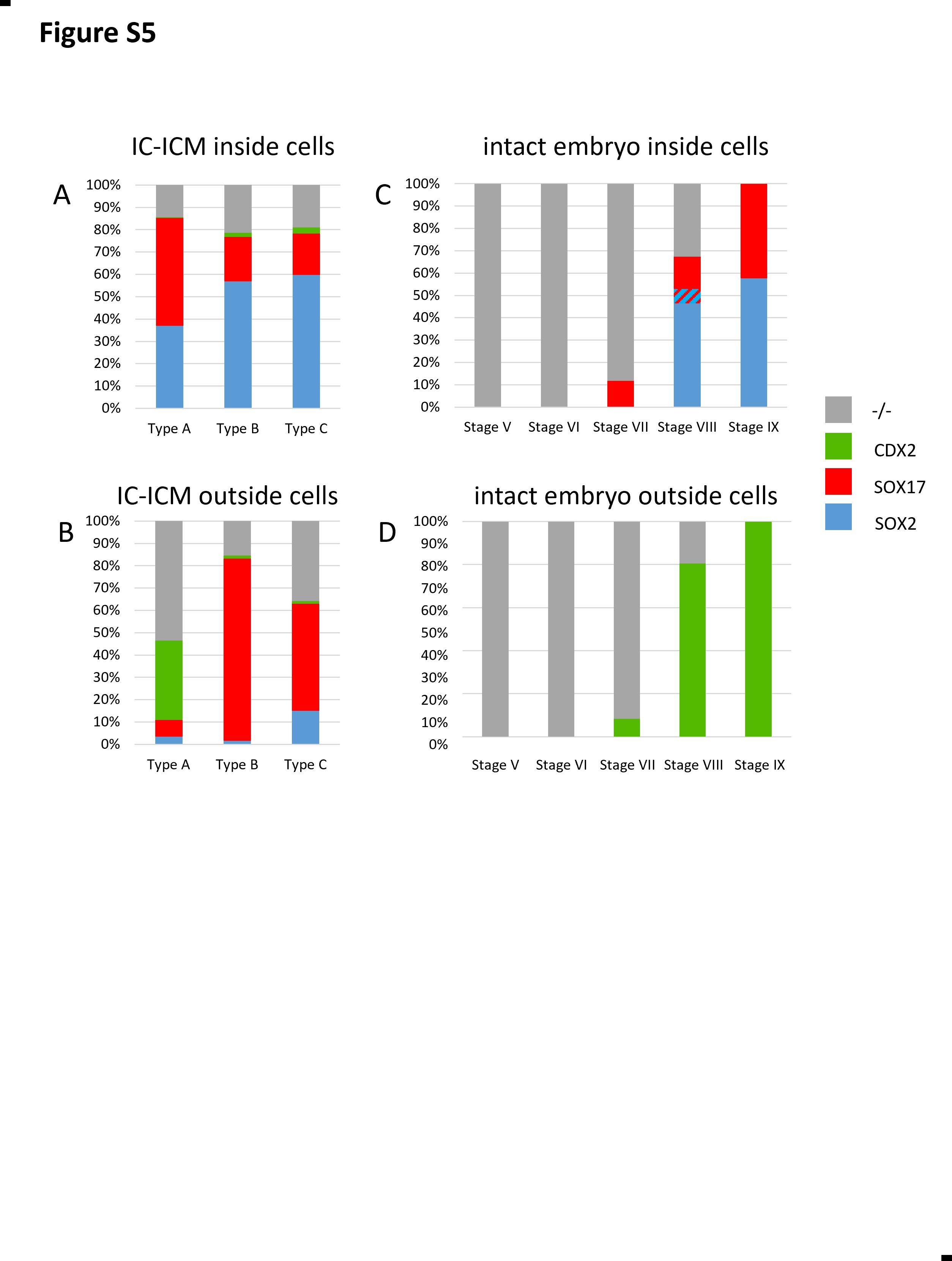

Supplement: Fig_S5_ioaf157 [file fig_s5_ioaf157.jpeg]

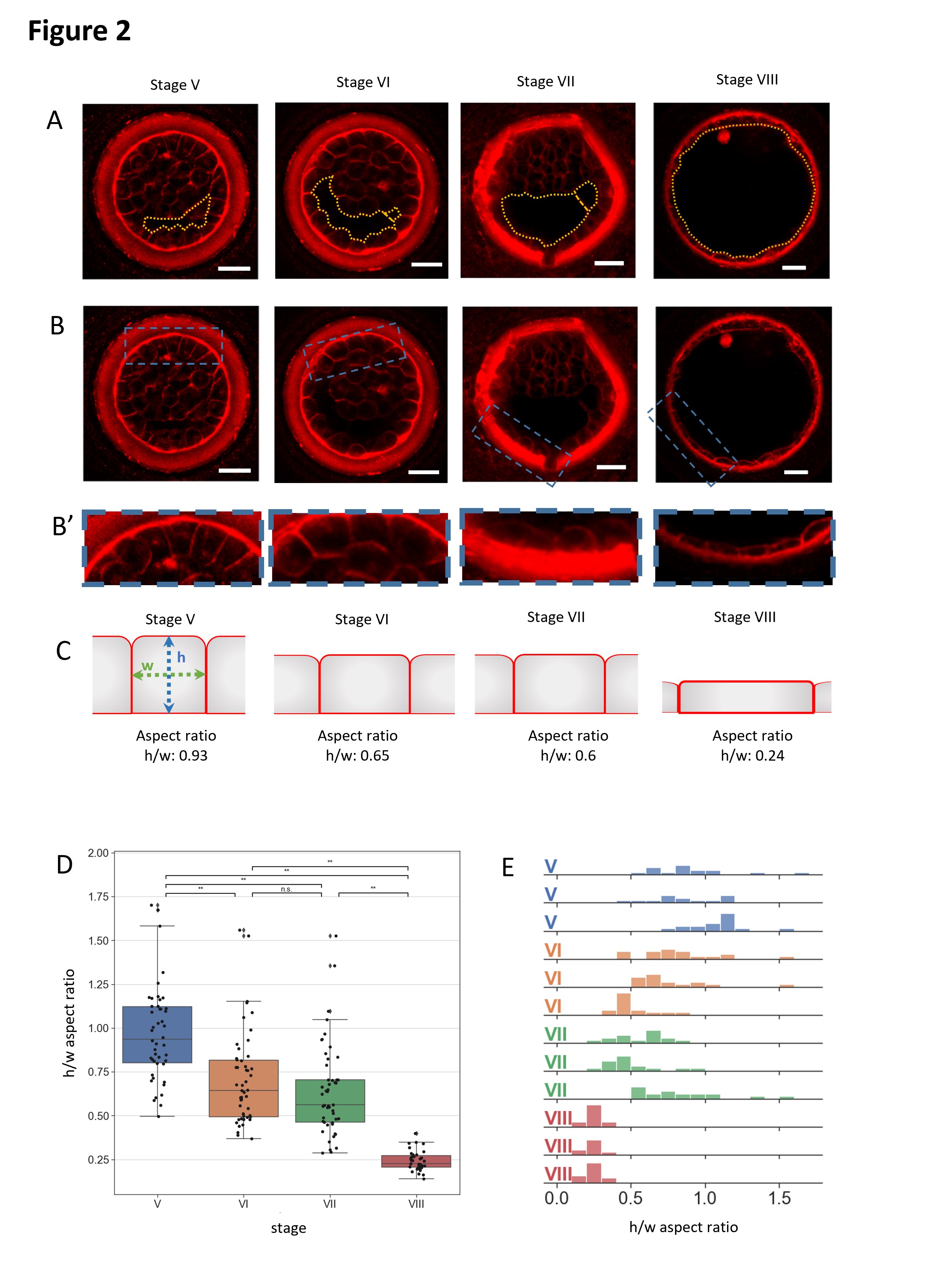

Supplement: Fig_2_resub_ioaf157 [file fig_2_resub_ioaf157.jpeg]
