# Supplementary material for: Stage-sensitive potential of isolated rabbit ICM to differentiate into extraembryonic lineages
Source: Biol Reprod. 2025 Jul 22;113(5):1102–20. doi: 10.1093/biolre/ioaf157 (PMC12621310; doi:10.1093/biolre/ioaf157)
Supplement: Supplementary_Figure_caption_ioaf157 [file supplementary_figure_caption_ioaf157.docx]

**Figure S1. (A) Confirmation of immunosurgery efficiency.** Live images of intact blastocysts stages VI, VII and VIII with TE labelled with multifluorescent (red and green) microspheres (blastocyst cavity partially collapsed following embryonic coats removal) (top) and isolated ICMs of the same blastocysts immediately following immunosurgery (bottom). Scale bar- 50 µm

**Figure S2.** Cytoplasmic bridges connect emerging ICM and TE during early stages of blastocyst cavity expansion. Selected still images of 4 different sample embryos from the Primo Vision time-lapse imaging system. Arrowheads highlight cytoplasmic bridges.

**Figure S3.** Expression levels of (A) CDX2, (B) GATA3 and (C) OCT4 mRNA in rabbit in vivo embryos at consecutive stages of development (E1.0 to E6.0) (n=24 embryos per stage). Differences in expression levels between consecutive developmental stages were tested using the Kruskal-Wallis test, and subsequent pairwise comparisons were conducted using the Conover test with a two-stage FDR correction (* for p<0.05, ** for p<0.005). Error bars represent standard error of the mean (SEM).

**Figure S4.** ICM-to-TE GATA3 fluorescence intensity ratio at stages VI-VIII. Kruskal-Wallis test (* for p<0.05). Error bars represent standard error of the mean (SEM). N cells: Stage VI = 308, Stage VII = 361, Stage VIII = 729

**Figure S5.** Comparison of composition of inside (A) and outside (B) compartment in IC-ICMs (classified according to the geometry of recavitation) vs inside (C) and outside (D) compartment in intact embryos. A and B identical to Fig. 8C; C based on data from Piliszek et al., 2017; D replotted from Fig. 3. Green – CDX2; Blue – SOX2; Red – SOX17; Grey – triple-negative cells
