# Supplementary material for: Stage-sensitive potential of isolated rabbit ICM to differentiate into extraembryonic lineages
Source: Biol Reprod. 2025 Jul 22;113(5):1102–20. doi: 10.1093/biolre/ioaf157 (PMC12621310; doi:10.1093/biolre/ioaf157)
Supplement: Supplementary_Movie_caption_ioaf157 [file supplementary_movie_caption_ioaf157.docx]

**Movie S1. In vitro development of rabbit blastocyst.** Time-lapse bright field movie of a rabbit embryo, fertilised in vivo, recovered at E3.0 late morula stage, and subsequently cultured in vitro under PrimoVision system.

**Movie S2. Type A “blastocyst” IC-ICM cavitation**. Time-lapse bright field movie of a rabbit IC-ICM cultured in vitro under PrimoVision system, developing a type A cavity geometry.

**Movie S3. Type B “halo” IC-ICM cavitation**. Time-lapse bright field movie of a rabbit IC-ICM cultured in vitro under PrimoVision system, developing a type B cavity geometry.
